# Clinicopathological Spectrum of EBV-Related Primary Splenic Tumors Identified by Splenectomy: A Case Series

**DOI:** 10.3390/diagnostics16020333

**Published:** 2026-01-20

**Authors:** Minju Kim, Byeong Gwan Noh, Myunghee Yoon, Hyung Il Seo, Myeong Hun Oh, Young Mok Park, Suk Kim, Seung Baek Hong, Kyung Un Choi

**Affiliations:** 1 Division of HBP Surgery and Transplantation, Department of Surgery, Biomedical Research Institute, Pusan National University Hospital, Pusan National University School of Medicine, 179 Gudeok-ro, Seo-gu, Busan 49241, Republic of Korea; minju.kim712@gmail.com (M.K.); ymh@pusan.ac.kr (M.Y.); ohmyunghun@naver.com (M.H.O.);; 2 Department of Radiology, Biomedical Research Institute, Pusan National University Hospital, Pusan National University School of Medicine, Busan 49241, Republic of Korea; 3 Department of Pathology, Biomedical Research Institute, Pusan National University Hospital, Pusan National University School of Medicine, Busan 49241, Republic of Korea

**Keywords:** Epstein–Barr virus, spleen, splenectomy, diffuse large B-cell lymphoma, inflammatory pseudotumor, follicular dendritic cell sarcoma

## Abstract

**Background:** Epstein–Barr virus (EBV)-related primary splenic tumors are exceptionally rare and encompass a heterogeneous group of entities, including inflammatory pseudotumor (IPT), IPT-like follicular dendritic cell (FDC) tumors or sarcomas, and EBV-positive diffuse large B-cell lymphoma (DLBCL). Because clinical presentation and imaging findings are often nonspecific, establishing a definitive diagnosis remains challenging and frequently necessitates splenectomy for histopathologic confirmation. **Methods:** We retrospectively reviewed patients who underwent laparoscopic splenectomy for suspected primary splenic lesions at a single tertiary institution between June 2014 and August 2025. Among 67 patients, five consecutive patients were pathologically confirmed as EBV-related primary splenic tumors. Clinical characteristics, imaging features, histopathologic and immunophenotypic findings, EBV in situ hybridization results, treatment, and follow-up outcomes were analyzed. **Results:** This case series comprised four spindle cell–predominant EBV-related tumors (IPT or IPT-like FDC tumors/sarcomas) and one EBV-positive DLBCL. All patients presented with splenic masses that could not be definitively characterized by preoperative imaging alone and therefore required splenectomy. EBV in situ hybridization was positive in tumor cells in all cases. Patients with non-lymphomatous tumors achieved durable disease control following splenectomy alone, with disease-free survival of up to five years. In contrast, the patient with EBV-positive DLBCL required postoperative systemic immunochemotherapy. **Conclusions:** EBV-related primary splenic tumors represent a diagnostically challenging and clinically diverse disease spectrum. This case series highlights the pivotal role of splenectomy in establishing definitive diagnosis and guiding subsequent management, particularly for isolated splenic lesions with indeterminate imaging findings.

## 1. Introduction

Primary splenic tumors associated with Epstein–Barr virus (EBV) are uncommon. Most published reports are single-case descriptions of tumors classified as inflammatory pseudotumor (IPT), IPT-like follicular dendritic cell (FDC) sarcoma, or EBV-positive diffuse large B-cell lymphoma (DLBCL) [1,2,3]. Because of the limited number of cases, their clinical course and pathological variety are not well established. EBV-positive IPT-like FDC sarcoma has been recognized as a distinct entity, often arising in the spleen or liver, with generally indolent behavior [2,4]. EBV-associated IPT lacking FDC marker expression has also been seen, raising uncertainty about whether some reflect reactive rather than truly neoplastic processes [3]. On the more aggressive side, primary splenic EBV-positive DLBCL is very rare but significant, as it demands systemic therapy in addition to surgery [5,6]. Preoperative diagnosis is difficult, since clinical symptoms and imaging lack specificity. Splenectomy is often needed not just for diagnosis but also may offer therapeutic benefit in non-lymphomatous disease. Here, we present a case series from our institution spanning June 2014 to August 2025, covering five EBV-related splenic tumors. Our aim is to compare clinical, radiologic, and pathologic features across distinct EBV-driven tumor subtypes, to better define their spectrum and clarify the role of splenectomy in diagnosis and management.

## 2. Methods

### 2.1. Patient Selection and Data Collection

This retrospective study was conducted at Pusan National University Hospital between June 2014 and August 2025. During this period, 67 patients underwent laparoscopic splenectomy for suspected primary splenic lesions. Primary splenic lesions were defined as splenic masses identified on preoperative imaging in the absence of a known dominant primary malignancy or systemic lymphoproliferative disease at the time of diagnosis. Patients with splenic involvement secondary to disseminated lymphoma, metastatic disease, infectious conditions, or traumatic injury were excluded. Of these patients, five consecutive cases were pathologically confirmed as Epstein–Barr virus (EBV)-related primary splenic tumors and were included in this case series. Diagnosis was established through histopathologic examination combined with Epstein–Barr-virus-encoded RNA in situ hybridization (EBER-ISH). Clinical characteristics, laboratory findings, radiologic features, operative details, pathologic results, adjuvant treatment, and follow-up outcomes were retrospectively reviewed using electronic medical records and are summarized in Table 1.

**Table 1:** Clinical, radiologic, and treatment characteristics of patients with EBV-related primary splenic tumors.

Case	Age/Sex	Clinical Features	CBC	LDH	Lesion Size (cm)	CT Findings	MRI/PET Distinctive Feature	Treatment	Outcome
1	69/F	Incidental finding on health screening	Normal	Normal	8.5 × 7.5 × 7.4 cm	Well-Circumscribed enhancing splenic mass with splenomegaly	PET: FDG uptake greater than liver (Deauville score 4)	Splenectomy	NED at 6 months
2	83/F	Detected during FU of cecal SMT	Normal	Normal	4.1 × 3.4 × 1.7 cm	Hypodense splenic lesion, abutting left kidney	MRI: delayed prolonged enhancement	Splenectomy	NED at 2 years
3	76/M	Incidental finding on health screening	Normal	Normal	5.1 × 5.0 × 3.2 cm	Heterogeneous enhancing splenic mass	MRI: heterogeneous enhancement; subtle T2 high SI compared with spleen	Splenectomy	NED at 5 years
4	69/M	Incidental finding on health screening	Normal	Normal	5.2 × 4.0 × 1.6 cm	Enhancing splenic mass	MRI: heterogeneous delayed prolonged enhancement with central scar	Splenectomy	NED at 5 years
5	88/F	Detected during evaluation for fever	Mild anemia	Elevated	Mutiple, 9.5 × 6.4 × 3.4 cm in the largest size	Multiple low density lesions	PET: hypermetabolic lesions	Splenectomy + R-CHOP	NED at 6 months; subsequently lost to FU

Abbreviations: CBC, complete blood count; LDH, lactate dehydrogenase; CT, computed tomography; MRI, magnetic resonance imaging; PET, positron emission tomography; EBV, Epstein–Barr virus; SMT, submucosal tumor; FU, follow-up; NED, no evidence of disease; SI, signal intensity.

### 2.2. Surgical Procedure

The indication for splenectomy was determined through multidisciplinary discussion and included radiologic suspicion of malignancy, progressive enlargement of the splenic lesion, abnormal metabolic uptake on positron emission tomography, or failure to achieve a definitive diagnosis using noninvasive modalities. All procedures were performed using a standard four-port laparoscopic technique, and the spleen was retrieved intact in all cases to allow for comprehensive pathological assessment.

### 2.3. Histopathologic Evaluation and Classification

Histologic evaluation was performed using hematoxylin and eosin (H&E) staining, supplemented by a panel of immunohistochemical markers assessing spindle cell, follicular dendritic cell, and lymphoid differentiation. Epstein–Barr-virus-encoded RNA in situ hybridization (EBER-ISH) was routinely applied in all cases. Detailed immunophenotypic profiles for each patient are summarized in Table 2. For the purpose of this study, EBV-related primary splenic tumors were classified into three diagnostic categories: inflammatory pseudotumor (IPT), inflammatory pseudotumor-like follicular dendritic cell (FDC) tumor, and EBV-positive diffuse large B-cell lymphoma (DLBCL). IPT was defined as a lesion composed predominantly of spindle cells with prominent inflammatory infiltrates, lacking significant cytologic atypia and without definitive expression of follicular dendritic cell markers. IPT-like FDC tumor was diagnosed when spindle or epithelioid tumor cells were associated with an inflammatory background and demonstrated evidence of follicular dendritic cell differentiation, indicated by immunoreactivity for CD21 and/or CD23 in conjunction with EBV positivity. EBV-positive DLBCL was diagnosed based on diffuse proliferation of atypical lymphoid cells expressing B-cell markers with EBER positivity. In cases showing equivocal or partial expression of follicular dendritic cell markers, diagnostic classification was determined based on the overall histomorphologic features, EBV status, and exclusion of alternative diagnoses, reflecting the recognized histologic spectrum of EBV-associated splenic tumors.

**Table 2:** Pathologic and immunophenotypic spectrum of primary splenic tumors with EBV positivity.

Case	Age/Sex	Pathologic Diagnosis	EBV-ISH	Key Morphology	Myofibroblastic Markers (SMA/Desmin)	FDC Markers (CD21, CD23, CD35)	B/T Cell Phenotype	Ki-67 (PI)	Distinctive Features
1	69/F	Inflammatory follicular dendritic cell (FDC) sarcoma, granulomatous variant	Positive	Spindle cell proliferation with granulomatous inflammation	SMA focal +, Desmin focal +	CD21/CD23 focal +, CD35 NA	Tumor −, background CD3+/CD20+	20–30%	Granulomatous inflammation; focal S100/EMA/CD68 positivity
2	83/F	Inflammatory pseudotumor (without FDC marker expression)	Positive	Spindle cell proliferation with inflammatory infiltrates	SMA +, Desmin −	CD21/CD23 −, CD35 NA	Tumor −, reactive CD3+/CD20+	~10%	Myofibroblastic proliferation; ALK−
3	76/M	Inflammatory pseudotumor-like dendritic cell sarcoma	Positive	Spindle cells, borderline atypia	SMA +, Desmin NA	CD21/CD23 focal +, CD35 NA	Tumor −, background CD3+/CD20+	NA	Increased IgG4+ plasma cells; ALK−
4	69/M	Inflammatory pseudotumor-like FDC tumor (two nodules)	Positive	Spindle/epithelioid cells, multinodular pattern	SMA +, Desmin NA	CD21 equivocal, CD23 −, CD35 NA	Tumor −, background CD3+/CD20+	20–30%	Multifocal nodules (1.3 cm, 5.2 cm); polyclonal plasma cells
5	88/F	Diffuse large B-cell lymphoma (DLBCL)	Positive	Sheets of large atypical lymphoid cells	SMA −, Desmin −	CD21/CD23 −, CD35 NA	Tumor: CD20+/CD79a+ /Bcl2+, CD3−	>80%	High-grade lymphoma phenotype

### 2.4. Ethics Statement

This study was approved by the Institutional Review Board of the Clinical Trial Center at Pusan National University Hospital (IRB No. 2509-012-154) and conducted in accordance with the Declaration of Helsinki. Written informed consent was obtained from all patients for publication of clinical information and imaging data.

## 3. Results

### 3.1. Patient Characteristics and Overview

**Figure 1:**
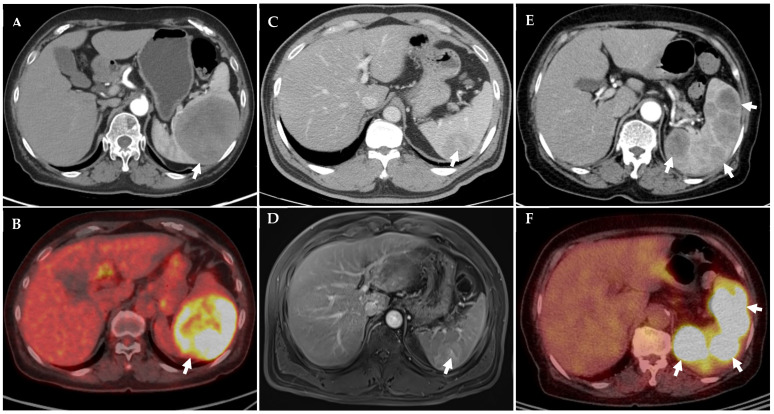
Multimodality imaging features of EBV-related primary splenic tumors. (**A**) Case 1, CT: well-circumscribed enhancing splenic mass measuring 8.5 × 7.5 cm (white arrow). (**B**) Case 1, PET/CT: FDG uptake greater than the liver, Deauville score 4 (white arrow). (**C**) Case 4, CT: enhancing splenic lesion with central stellate scar, measuring 5.2 × 4.0 cm (white arrow). (**D**) Case 4, MRI (delayed phase): heterogeneous prolonged enhancement with central scar (white arrow). (**E**) Case 5, CT: multiple hypodense splenic lesions, largest measuring 9.5 × 6.4 cm (white arrows). (**F**) Case 5, PET/CT: hypermetabolic activity confined to the spleen (white arrows).

**Figure 2:**
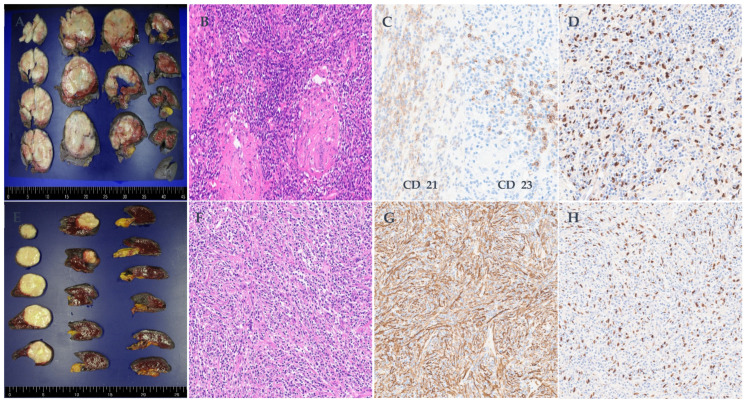
Histopathologic spectrum of EBV-related splenic tumors. Case 1: (**A**) Gross finding showing a well-circumscribed, capsulated yellow–white solid mass (8.5 × 7.5 × 7.4 cm). (**B**) H&E stain (×200) demonstrates spindle cell proliferation with lymphoplasmacytic infiltrates and granulomatous inflammation. (**C**) CD21/CD23 immunostain (×200) highlights focal positivity in scattered spindle cells. (**D**) EBV-ISH (×200) shows strong nuclear positivity in spindle cells. Case 2: (**E**) Gross finding showing an encapsulated yellow–white solid mass (4.1 × 3.4 × 1.7 cm). (**F**) H&E stain (×200) demonstrates myofibroblastic spindle cell proliferation with mixed inflammatory infiltrates. (**G**) SMA immunostain (×200) shows diffuse cytoplasmic positivity in spindle cells. (**H**) EBV-ISH (×200) highlights nuclear positivity in spindle cells.

Between 2014 and 2025, five patients were diagnosed with EBV-related primary splenic tumors following laparoscopic splenectomy. This case series included three women and two men, with a median age of 76 years (range, 69–88 years). The clinical characteristics, laboratory findings, imaging features, treatments, and outcomes are summarized in Table 1, while detailed pathologic and immunophenotypic profiles are provided in Table 2. Representative radiologic findings are shown in Figure 1A–F, and histopathologic features are illustrated in Figure 2A–H. All patients presented with splenic lesions detected incidentally or during evaluation for unrelated symptoms. Four patients had solitary splenic masses, whereas one patient exhibited multiple splenic lesions. Preoperative laboratory findings were largely unremarkable, except for mild anemia and elevated lactate dehydrogenase in one patient. Radiologic evaluation demonstrated heterogeneous or progressive enhancement patterns on computed tomography and magnetic resonance imaging, as well as increased metabolic activity on positron emission tomography in selected cases. Pathologically, the spectrum of EBV-related splenic tumors in this series comprised three inflammatory pseudotumor (IPT) or IPT-like follicular dendritic cell (FDC) lesions, one granulomatous variant of inflammatory FDC sarcoma, and one EBV-positive diffuse large B-cell lymphoma (DLBCL). None of the patients had a documented history of underlying inflammatory or autoimmune disease or prior exposure to immunosuppressive agents. There was also no clinical or radiologic evidence of peripheral blood or bone marrow involvement at presentation.

All cases demonstrated EBV positivity by EBV-ISH. Spindle cell lesions, including inflammatory pseudotumor and IPT-like FDC tumors/sarcomas, showed variable SMA expression with absent or only focal FDC marker reactivity, while the granulomatous variant was characterized by granulomatous inflammation and focal S100/EMA/CD68 staining. In all IPT-like cases, tumor cells were negative for B- and T-cell markers, with such expression limited to background lymphocytes. The DLBCL case was distinguished by diffuse CD20/CD79a positivity, lack of T-cell marker expression, and a markedly elevated proliferative index (Ki-67 > 80%). Abbreviations: ALK, anaplastic lymphoma kinase; Bcl2, B-cell lymphoma 2; CD, cluster of differentiation; DLBCL, diffuse large B-cell lymphoma; EBV-ISH, Epstein–Barr virus in situ hybridization; EMA, epithelial membrane antigen; FDC, follicular dendritic cell; H&E, hematoxylin and eosin; IgG4, immunoglobulin G4; IHC, immunohistochemistry; Ki-67, proliferation index marker; NA, not available; SMA, smooth muscle actin.

### 3.2. Case Presentations

In most cases, splenic lesions were incidentally detected during health screening or evaluation for unrelated conditions; however, splenectomy was pursued because imaging findings were indeterminate, interval growth was observed, or malignancy could not be excluded, necessitating surgical intervention for definitive diagnosis and management.

#### 3.2.1. Case 1

A 69-year-old woman was incidentally found to have an 8.5 × 7.5 cm splenic mass during routine health screening. Computed tomography demonstrated a well-circumscribed lesion with progressive enhancement, and positron emission tomography revealed fluorodeoxyglucose uptake greater than that of the liver (Deauville score 4). The histopathologic examination confirmed an inflammatory FDC sarcoma, granulomatous variant, characterized by spindle cell proliferation with granulomatous inflammation, focal CD21 and CD23 immunoreactivity, and strong EBV-ISH positivity. The patient remains without evidence of disease at six months of follow-up and continues under active surveillance without additional therapy.

#### 3.2.2. Case 2

An 83-year-old woman undergoing follow-up for a cecal submucosal lesion, which was biopsied and showed no evidence of lymphoproliferative disease, showed interval growth of a splenic mass from 0.8 cm to 4.1 × 3.4 cm. Magnetic resonance imaging demonstrated delayed and prolonged enhancement. Splenectomy revealed an EBV-associated inflammatory pseudotumor without follicular dendritic cell marker expression. Histologic findings included myofibroblastic spindle cells with smooth muscle actin positivity and EBV-ISH reactivity. The patient has remained disease-free for two years following surgery.

#### 3.2.3. Case 3

A 76-year-old man was incidentally found to have a 5.1 × 5.0 cm splenic lesion. Computed tomography showed heterogeneous enhancement, and magnetic resonance imaging demonstrated subtle T2 hyperintensity relative to the surrounding splenic parenchyma. Pathologic evaluation established the diagnosis of an IPT-like follicular dendritic cell tumor. The patient has remained alive without evidence of recurrence for five years after splenectomy.

#### 3.2.4. Case 4

A 69-year-old man presented with a 5.2 × 4.0 cm splenic lesion detected on imaging. Computed tomography demonstrated heterogeneous enhancement with a central stellate scar, while delayed-phase magnetic resonance imaging showed persistent enhancement. Histologic examination revealed features consistent with an IPT-like FDC tumor with a multinodular growth pattern. The patient remains free of disease at five years of follow-up.

#### 3.2.5. Case 5

An 88-year-old woman presented with fever, and computed tomography revealed multiple low-density splenic lesions, the largest measuring 9.5 × 6.4 cm. Positron emission tomography/computed tomography demonstrated hypermetabolic activity confined to the spleen. Splenectomy confirmed EBV-positive diffuse large B-cell lymphoma. Postoperative imaging demonstrated complete resolution of splenic disease. The patient subsequently received three cycles of R-CHOP chemotherapy, which was discontinued due to advanced age and limited treatment adherence. She remained disease-free at six months of follow-up but was subsequently lost to surveillance.

### 3.3. Postoperative Outcomes and Follow-Up

All patients underwent successful laparoscopic splenectomy without perioperative mortality. No major postoperative complications were observed, and no cases of overwhelming postsplenectomy infection occurred during the follow-up period. The median duration of follow-up for this case series was 24 months (range, 6–60 months). Patients with IPT or IPT-like FDC tumors demonstrated favorable outcomes following splenectomy alone, with no evidence of recurrence or disease progression. The patient with EBV-positive DLBCL achieved complete radiologic remission after splenectomy and limited adjuvant chemotherapy. At the last available follow-up, all patients were alive, and no disease-related mortality was documented.

## 4. Discussion

Primary EBV-related tumors of the spleen are exceedingly rare entities and encompass a wide histopathological spectrum. The present case series highlights this heterogeneity by capturing entities ranging from EBV-associated inflammatory pseudotumor (IPT) and IPT-like follicular dendritic cell (FDC) tumors to EBV-positive diffuse large B-cell lymphoma (DLBCL). Although unified by EBV positivity, these lesions differed substantially in morphology, immunophenotype, and clinical behavior, underscoring the diagnostic and therapeutic challenges they pose in routine practice.

### 4.1. Diagnostic Challenges and the Role of Imaging

Preoperative differentiation between EBV-related splenic tumors remains difficult because clinical manifestations are often absent or nonspecific and radiologic findings substantially overlap. In our series, most lesions were detected incidentally during routine examinations, consistent with prior reports emphasizing the limited diagnostic specificity of imaging in this setting [7,8,9]. Contrast-enhanced CT reliably identified splenic masses but demonstrated variable enhancement patterns that could not distinguish IPT-like lesions from lymphoma [7,8,9]. MRI provided additional, albeit still nonspecific, information: lesions frequently showed heterogeneous T2 signal intensity and delayed or prolonged enhancement, and in one case a central stellate scar was present—an imaging feature that can also be encountered in sclerosing angiomatoid nodular transformation (SANT), which typically shows spoke-wheel enhancement and relative T2 hypointensity [7,8,9]. PET/CT was most informative in the lymphomatous case, in which EBV-positive DLBCL exhibited avid FDG uptake confined to the spleen (Deauville score 4). This observation aligns with previous evidence that PET/CT improves detection and response assessment in splenic lymphomas, while remaining less specific for benign or low-grade spindle cell lesions [7,8,9]. Taken together, CT, MRI, and PET/CT serve as useful adjuncts but rarely establish a definitive diagnosis. Although these imaging findings are not diagnostic, certain tendencies—such as delayed or prolonged enhancement, the presence of a central stellate scar, and relatively lower or heterogeneous FDG uptake—may provide supportive clues when interpreted in the appropriate clinical context. Consequently, splenectomy continues to play a pivotal role by providing sufficient tissue for histopathologic evaluation, EBV-encoded RNA in situ hybridization, and comprehensive immunohistochemical profiling.

### 4.2. Histopathological Spectrum and EBV Association

Our findings support the concept that EBV-related splenic tumors represent a pathologic continuum rather than discrete, unrelated entities. Accordingly, in cases with focal or equivocal expression of follicular dendritic cell markers, diagnostic classification should rely on integrated clinicopathologic assessment rather than any single immunohistochemical marker. IPT-like FDC sarcoma is now recognized as an EBV-driven neoplasm with a predilection for the spleen and liver and generally follows an indolent clinical course. Diagnosis depends on EBER positivity in spindle or ovoid tumor cells, at least focal expression of FDC markers (CD21, CD23, CD35), frequent co-expression of smooth muscle actin, and consistent ALK negativity—features that help distinguish it from inflammatory myofibroblastic tumor and EBV-negative conventional FDC sarcoma [4,10]. In contrast, EBV-associated IPT lacking FDC marker expression is characterized by EBER-positive myofibroblastic spindle cells without CD21/CD23/CD35 or ALK reactivity. Some cases demonstrate increased IgG4-positive plasma cells, supporting the notion that these lesions occupy an intermediate zone between reactive inflammatory processes and true neoplasia [3,11,12]. At the malignant end of the spectrum, EBV-positive DLBCL of the spleen is exceptionally rare but clinically consequential, consisting of EBER-positive, CD20/CD79a-positive B cells with a high proliferative index and necessitating systemic therapy rather than surgery alone [5,6,13]. Notably, our series included a granulomatous variant of inflammatory FDC sarcoma, characterized by prominent granulomatous inflammation admixed with EBV-positive FDC-lineage cells—a form that has been reported only sporadically in the literature [14]. Viewed collectively, these tumors align with recently proposed categories of stroma-derived splenic neoplasms, which emphasize shared microenvironmental and viral drivers despite divergent morphology and immunophenotype [15]. In all five cases, EBV-ISH positivity in tumor cells confirmed EBV as the unifying pathogenic factor and enabled precise subclassification when combined with targeted immunohistochemistry.

### 4.3. Management Implications

This case series underscores the dual diagnostic and therapeutic role of splenectomy in EBV-related splenic tumors. For non-lymphomatous lesions, including IPT and IPT-like FDC tumors or sarcomas, splenectomy alone resulted in durable disease control without the need for adjuvant therapy, consistent with prior surgical reports advocating resection as definitive management [16]. When feasible, laparoscopic splenectomy offers the advantages of minimal invasiveness while ensuring intact specimen retrieval for accurate pathologic assessment. Given the lifelong risk of overwhelming postsplenectomy infection, perioperative vaccination and infection-prevention counseling should be integral components of surgical care. By contrast, EBV-positive DLBCL of the spleen adheres to established lymphoma treatment principles. In such cases, splenectomy primarily serves diagnostic and staging purposes, whereas systemic immunochemotherapy constitutes the cornerstone of treatment. Rituximab-based regimens such as R-CHOP, with PET/CT-guided response assessment, remain standard, although abbreviated or attenuated protocols may be considered in very elderly or frail patients [17]. These distinctions highlight the importance of early multidisciplinary collaboration between surgeons, radiologists, pathologists, and hematologist–oncologists to ensure timely diagnosis and guideline-concordant therapy. Practically, when imaging cannot exclude lymphoma or reliably characterize an isolated splenic mass, splenectomy remains the most reliable means of obtaining adequate tissue for definitive diagnosis. Conversely, in patients with disseminated disease suggestive of systemic lymphoma, image-guided biopsy at a more accessible site may be preferred, reserving splenectomy for unresolved diagnostic uncertainty or symptomatic splenomegaly.

### 4.4. Comparison with Prior Literature

Most published data on EBV-related splenic tumors consist of isolated case reports or very small series focused on individual entities, such as IPT-like FDC sarcoma, EBV-associated IPT, or EBV-positive DLBCL [1,4,13]. While these reports underscore the rarity and variable behavior of such tumors, they rarely encompass the full clinicopathological spectrum within a single institutional experience. Our case series adds to the literature by documenting all three major categories within one center, enabling direct comparison of clinical presentation, imaging characteristics, immunophenotypic features, and outcomes. The indolent course and sustained disease-free survival observed in our IPT and IPT-like FDC cases parallel prior observations supporting favorable prognosis after splenectomy alone [11,12]. In contrast, the need for systemic therapy in EBV-positive DLBCL mirrors earlier reports emphasizing the limitations of surgery as definitive treatment in this context [5,6,13]. The inclusion of a granulomatous variant of inflammatory FDC sarcoma further expands the limited body of published experience with this rare subtype [14]. Collectively, our findings reinforce and extend existing knowledge, supporting the view that EBV-related splenic tumors constitute a biologically linked spectrum unified by viral pathogenesis despite marked phenotypic diversity [15].

### 4.5. Limitations and Future Perspectives

Several limitations warrant consideration. The retrospective, single-center design and small sample size reflect the rarity of EBV-related splenic tumors and limit the generalizability of our observations. The advanced age of the study population may also have influenced tumor biology, immune surveillance, and clinical outcomes, further limiting the generalizability of the findings. Molecular analyses, such as EBV clonality assessment or next-generation sequencing, were not performed and could have provided deeper insight into tumor biology and potential therapeutic targets; likewise, circulating EBV-DNA levels in serum or plasma were not assessed and may have offered complementary information for risk stratification and preoperative evaluation. In addition, follow-up duration was limited in some patients, precluding definitive conclusions regarding long-term outcomes. Nevertheless, by systematically comparing the clinical, radiologic, and pathologic features across the EBV-related splenic tumor spectrum, this case series provides practical insights for diagnosis and management. Future multicenter studies with pooled cohorts and integrated molecular profiling will be essential to refine prognostic stratification and optimize evidence-based treatment strategies.

## 5. Conclusions

EBV-related primary splenic tumors are exceptionally rare and heterogeneous. This five-case series highlights their diagnostic challenges and emphasizes the pivotal role of splenectomy, offering practical insights for managing unexplained splenic masses.

## Data Availability

The original contributions presented in this study are included in the article. Further inquiries can be directed to the corresponding author.

## References

[1] Pagliuca F., Ronchi A., Auricchio A., Lieto E., Franco R. (2022). Inflammatory pseudotumor-like follicular/fibroblastic dendritic cell sarcoma: Focus on immunohistochemical profile and association with Epstein-Barr virus. Infect. Agent. Cancer.

[2] Cheuk W., Chan J.K., Shek T.W., Chang J.H., Tsou M.H., Yuen N.W., Ng W.-F.M., Chan A.C.L.M., Prat J. (2001). Inflammatory pseudotumor-like follicular dendritic cell tumor: A distinctive low-grade malignant intra-abdominal neoplasm with consistent Epstein-Barr virus association. Am. J. Surg. Pathol..

[3] Abe K., Kitago M., Matsuda S., Shinoda M., Yagi H., Abe Y., Oshima G., Hori S., Endo Y., Yokose T. (2022). Epstein-Barr virus-associated inflammatory pseudotumor variant of follicular dendritic cell sarcoma of the liver: A case report and review of the literature. Surg. Case Rep..

[4] Jin J., Zhu X., Wan Y., Shi Y. (2024). Epstein-Barr virus (EBV)-positive inflammatory pseudotumor-like follicular dendritic cell sarcoma presenting as thrombocytopenia: A case report and literature review. Heliyon.

[5] Seijari M.N., Kaspo S., Alshurafa A., Elfaieg A., Elkourashy S.A. (2024). Primary splenic diffuse large B-cell lymphoma: A case report and literature review of a rare condition. Case Rep. Oncol..

[6] Shi Y., Han Y., Yang J., Liu P., He X., Zhang C., Zhou S., Zhou L., Qin Y., Song Y. (2019). Clinical features and outcomes of diffuse large B-cell lymphoma based on nodal or extranodal primary sites of origin: Analysis of 1,085 WHO classified cases in a single institution in China. Chin. J. Cancer Res..

[7] Jang S., Kim J.H., Hur B.Y., Ahn S.J., Joo I., Kim M.J., Han J.K. (2018). Role of CT in differentiating malignant focal splenic lesions. Korean J. Radiol..

[8] Kim N., Auerbach A., Manning M.A. (2022). Algorithmic approach to the splenic lesion based on radiologic-pathologic correlation. Radiographics.

[9] Valizadeh P., Jannatdoust P., Tahamtan M., Ghorani H., Dorcheh S.S., Farnoud K., Salahshour F. (2024). Diagnostic performance of different imaging modalities for splenic malignancies: A comparative meta-analysis. Eur. J. Radiol. Open.

[10] Bruehl F.K., Azzato E., Durkin L., Farkas D.H., Hsi E.D., Ondrejka S.L. (2020). Inflammatory pseudotumor-like follicular/fibroblastic dendritic cell sarcomas of the spleen are EBV-associated and lack other commonly identifiable molecular alterations. Int. J. Surg. Pathol..

[11] Ge R., Liu C., Yin X., Chen J., Zhou X., Huang C., Yu W., Shen X. (2014). Clinicopathologic characteristics of inflammatory pseudotumor-like follicular dendritic cell sarcoma. Int. J. Clin. Exp. Pathol..

[12] Baber A., Legendre P., Palmic P., Lupo-Mansuet A., Burroni B., Azoulay C., Szwebel T.-A., Costedoat-Chalumeau N., Leroy K., Blons H. (2024). EBV-positive inflammatory follicular dendritic cell sarcoma of the spleen: Report of an aggressive form with molecular characterization. Int. J. Surg. Pathol..

[13] Bourbon E., Maucort-Boulch D., Fontaine J., Mauduit C., Sesques P., Safar V., Ferrant E., Golfier C., Ghergus D., Karlin L. (2021). Clinicopathological features and survival in EBV-positive diffuse large B-cell lymphoma not otherwise specified. Blood Adv..

[14] Nie C., Xie X., Li H., Li Y., Chen Z., Li Y., Li Z. (2024). Epstein-Barr virus-positive inflammatory follicular dendritic cell sarcoma with significant granuloma: Case report and literature review. Diagn. Pathol..

[15] Prasad A.S., Chua S.S., Ramani N.S., Shiralkar K.G., Shanbhogue K.P., Surabhi V.R. (2025). Stroma-derived neoplasms and pseudoneoplastic lesions of the spleen: A select review of pathologic and CT/MRI findings. Abdom. Radiol..

[16] Giovagnoni A., Giorgi C., Goteri G. (2005). Tumours of the spleen. Cancer Imaging.

[17] Tilly H., da Silva M.G., Vitolo U., Jack A., Meignan M., Lopez-Guillermo A., Walewski J., André M., Johnson P.W., Pfreundschuh M. (2015). Diffuse large B-cell lymphoma (DLBCL): ESMO Clinical Practice Guidelines for diagnosis, treatment and follow-up. Ann. Oncol..

